# Utilization of bull fertility-associated antigen to improve the quality of frozen bull semen

**DOI:** 10.14202/vetworld.2020.2112-2117

**Published:** 2020-10-09

**Authors:** Tri Wahyu Suprayogi, Hardijanto Hardijanto, Mas’ud Hariadi, Fedik Abdul Rantam, Win Darmanto

**Affiliations:** 1 Department of Veterinary Reproduction, Faculty of Veterinary Medicine, Universitas Airlangga, Surabaya 60115, Indonesia; 2 Department of Microbiology and Virology, Faculty of Veterinary Medicine, Universitas Airlangga, Surabaya 60115, Indonesia; 3 Department of Biology, Faculty of Science and Technology, Universitas Airlangga, Surabaya 60115, Indonesia

**Keywords:** fertility associated antigen, gland accessories bull, semen fertility

## Abstract

**Background and Aim:**

The implementation of artificial insemination (AI) is one of the strategies to use superior male semen optimally to improve the genetic quality of livestock. One of the factors that influence AI is a fertility-associated antigen (FAA). This research aimed to examine the effects of FAA extracted from the accessory sex glands of a bull from a slaughterhouse that was added in bull semen freezing medium to increase cattle (bull) fertilization.

**Materials and Methods:**

This research used a randomized complete block design. It consisted of two research phases, namely, explorative and experimental phases. The first phase involved determining the FAA molecular weight using the SDS-PAGE method, and the second phase consisted of laboratory and field testing, including testing the quality of frozen semen supplemented with FAA extracted from the accessory glands of a bull’s genital organ from a slaughterhouse with various doses (0, 5, 10, and 15 μg in every 200 million progressively motile spermatozoa).

**Results:**

The results showed that the percentages of bull sperm motility between the groups without and with the additional administration of FAA with a dose of 5 μg did not significantly differ. However, there was a difference between the groups without and with the additional administration of FAA with doses of 10 and 15 μg. After further testing, the highest percentage of sperm progressive motility occurred at a dose of 15 μg/200 million progressively motile spermatozoa (P3), which was equal to 2.59±46.88b (%).

**Conclusion:**

This research found that not all of the accessory glands (seminal vesicles) of bulls taken from the slaughterhouse contain the FAA. An FAA level between the accessory glands (seminal vesicles) of one cattle to another is different. The addition of the FAA protein from the accessory sex glands of a bull’s organ in cattle semen can improve fertility by increasing the percentage of viability, motility, intact plasma membrane of spermatozoa, and pregnancy rate of bulls and decreasing the sperm capacitation post-thawing.

## Introduction

The implementation of artificial insemination (AI) is one of the strategies to use superior male semen optimally to improve the genetic quality of livestock. However, the actual success rate is 65% [1]. Several factors influence the success of AI, for example, breed, season, semen origin, region, and inseminator [2].

The quality of semen origin plays a significant role in the incidence of pregnancy in female livestock. Other factors that affect the quality of semen include AI delivery system, heat detection and time of insemination, intrinsic factors, early embryonic losses, problem of management, insemination technique, and individual awareness [3]. Considering this phenomenon, it is necessary to have high fertility in the frozen semen. Hence, the ingredients that can improve fertility should be added to the semen that is about to be frozen. The development and research about the additional materials to improve the quality of frozen semen are still being conducted until now.

Fertility-associated antigen (FAA) is a specific protein that is produced by the accessory sex glands and secreted into the fluid that may bind heparin and accelerate sperm cell capacitation after ejaculation. Consequently, bull fertility is improved because it is able to increase the possibility when semen is deposited in the uterine body [3].

In this research, the isolation, identification, and characterization of the FAA from the accessory sex glands of a bull that has been cut as an additive material to increase the rate of *in vitro* and *in vivo* fertilization in freezing medium were conducted. The increasing rate of *in vitro* and *in vivo* fertilization can further enhance the fertility and productivity of livestock. This can be used as the basis for the development of the quality of frozen semen production in all national and regional AI centers.

## Materials and Methods

### Ethical approval

Ethical approval is not necessary for such type of study. However, all the animals were properly handled by the researchers. No animals were harmed during this research.

### Research period and location

The research was conducted from March to October 2014 in the Frozen Semen Laboratory for the extraction, dilution, and manufacture of frozen semen and the Laboratory of Molecular Biology for the extraction, characterization, and isolation of the FAA protein from bull semen. The testing of anti-FAA specificity was conducted in the Faculty of Veterinary Medicine, Universitas Airlangga. Sperm capacitation examination was conducted using fluorescence microscopy in the Central Laboratory of Biological Sciences and Animal Reproduction Laboratory of the Faculty of Animal Husbandry, Brawijaya University. Field testing was conducted in the Mustika CV cattle farm in Wonosalam, Jombang.

### Research method

This research consisted of two phases. The first phase was explorative, and the second was experimental. In this research, we used a randomized complete block design [4]. The first phase of the research involved determining the FAA molecular weight (MW) using the SDS-PAGE method that was confirmed by Western blotting with an anti-FAA monoclonal antibody (ABM Inc., no. Cat. Y123009). The determination of the FAA level with the Qubit^®^ fluorometer method was also conducted in the first phase. The second phase consisted of (i) the freezing-thawing protocol, (ii) addition of FAA, and (iii) testing to assess sperm motility, viability, intact plasma membrane (IPM), and capacitation as well as the quality of frozen semen supplemented with FAA extracted from the accessory glands of a bull’s genital organ from a slaughterhouse with various doses (0, 5, 10, and 15 μg in every 200 million progressively motile spermatozoa); there were 32 bulls used in this research. The *in vivo* fertility test of frozen semen by inseminating it to a cow was also conducted in the second phase.

### First phase of the research

The first phase of the research was an explorative research to determine the characteristics of the FAA proteins on the accessory sex glands of a bull from a slaughterhouse. Thus, there was no statistical test. The data obtained from this research were tabulated descriptively. The parameters measured in this phase were the MW of the protein of the accessory sex glands of the bull and the FAA MW and level.

### Second phase of the research

The second phase of the research was an experimental research to determine the potential of the FAA from the extraction of the accessory glands of a bull’s genital organ on the viability, motility, IPM, capacitation, and *in vivo* fertilization rate of bull’s sperm post-thawing.

### Statistical analysis

The data percentage of viability, motility, IPM (MPU), and capacitation was determined using the ANOVA test at 5% confidence level. If there were differences in univariate data, it was then continued with Duncan’s distance test, and the pregnancy rate of bulls was determined using the Chi-square test [5].

## Results and Discussion

### The effect of FAA on the motility (%) of bull sperm

The mean (±standard deviation) of the percentage of bull sperm motility post-thawing from various treatments is shown in Table-1.

**Table 1:** Mean (±Standard deviation) and F-test of the percentage of cow spermatozoa motility post-thawing on control group and treatment groups added with various doses of FAA.

Treatment	Mean of spermatozoa motility (%)±Standard deviation
P_0_	41.25^a^±2.32
P_1_	42.50^a^±2.67
P_2_	45.63^b^±3.20
P_3_	46.88^b^±2.59

P_0_=Control (without being added with FAA), P_1_=Added with FAA 5 μg/200 millions spz progressive motility, P_2_=Added with FAA 10 μg/200 millions spz progressive motility, P_3_=Added with FAA 15 μg/200 millions spz progressive motility. FAA=Fertility-associated antigen, a, b and c shows the significant difference

The percentage of sperm progressive motility is a significant indicator for determining the quality of semen; because of this progressive motility, the sperm can reach the infundibulum to penetrate into the ovum. Individual progressive motility is defined as the ability of the sperm to move forward individually. This progressive propulsion is required by sperms once they are in the female reproductive tract trying to reach the site of fertilization, especially when penetrating the zona pellucida of the ovum [6].

The results showed that the percentages of bull sperm motility between the groups without and with the additional administration of FAA with a dose of 5 μg did not differ. However, a difference between the groups without and with the additional administration of FAA with doses of 10 and 15 μg exists. After further testing, the highest percentage of sperm progressive motility occurred at a dose of 15 μg/200 million progressively motile spermatozoa, which was equal to 2.59±46.88b (%). This proved that the addition of the FAA with a dose of 15 μg may be the optimal dose of the FAA protein in improving autophosphorylation associated with the increase in sperm motility. According to Georgadaki *et al*. [5], the occurrence of tyrosine kinase phosphorylation in the principal piece of sperm is associated with sperm motility. The increase in tyrosine kinase phosphorylation will improve sperm motility hyperactivation in the process of penetration into the zona pellucida of the ovum. This is consistent with the result of the research conducted by Jin and Yang [7], stating that the factors that influence the AI include the occurrence of motility hyperactivation and zona pellucida reaction.

Based on the statistical test result, the groups without and with the additional administration of FAA with a dose of 5 μg did not significantly differ. This indicated that administering the FAA with a dose of 5 μg had not been able to bind the components of the plasma membrane of all sperms (200 million). Therefore, a number of sperms had unstable plasma membrane. The membrane instability caused several sperms to be unable to work upon thawing. Consequently, ATP was not produced, and many motile sperms occurred.

Sperm motility in both groups without and with the additional administration of FAA with a dose of 5 μg in semen before thawing did not increase. This was presumably because there were still several FAA receptors on the sperm membrane that was not bonded with exogenous FAA; hence, the signal transduction pathway to trigger an increase in tyrosine kinase phosphorylation did not occur, and tyrosine kinase phosphorylation was likely to remain the same. This proved that the FAA dose reduction did not increase the percentage of individual progressive motility. Garcia-Alvarez *et al*. [8] stated that the increase in membrane proteins will trigger the signal transduction pathway that affects the tyrosine kinases endogenous to bind the substrate (ATP) in the sperm. It resulted in an increase in tyrosine kinase phosphorylation production. However, when the content of the membrane proteins is low, there will not be enough tyrosine kinase endogenous to bind the substrate, resulting in less phosphorylation and absence of sperm motility.

### The effect of FAA on the viability (%) of bull sperm

The mean (±standard deviation) of the percentage of bull sperm viability post-thawing from various treatments is shown in Table-2. The ice formation process will result in membrane damage causing massive water molecule expenditure from the sperm, leading to an increase in the concentration of intracellular electrolytes and formation of ice crystals. When this happens continuously, the sperm will experience death. The damage that occurred during the process of cooling, freezing, and thawing especially in the cell membrane, either at the plasma membrane or mitochondrion, can also affect to the core [9].

**Table 2:** Mean (±Standard deviation) and F-test of the percentage of cow spermatozoa viability post-thawing on control group and treatment groups added with various doses of FAA.

Treatment	Mean of spermatozoa viability (%)±Standard deviation
P^0^	50.87^a^±2.92
P^1^	52.81^a^±2.71
P^2^	56.20^a^±3.28
P^3^	57.35^b^±2.89

P^0^=Control (without being added with FAA), P^1^=Added with FAA 5 μg/200 millions spz progressive motility, P^2^=Added with FAA 10 μg/200 millions spz progressive motility, P^3^=Added with FAA 15 μg/200 millions spz progressive motility. FAA=Fertility-associated antigen, a and b shows significant difference at p<0.05

The results showed that the sperm viability between the groups without and with the additional administration of FAA with a dose of 5 μg did not significantly differ. However, there was a difference between the groups without and with the additional administration of FAA with doses of 10 and 15 μg. After further testing, the highest percentage of sperm viability occurred in the group with the additional administration of FAA with a dose of 15 μg/200 million progressively motile spermatozoa (equal to 57.35±2.89b [%]) compared with that in the group without the additional administration of FAA (equal to 50.87±2.92) and the groups with the additional administration of FAA with doses of 5 and 10 μg (equal to 52.81±2.71 and 56.20±3.28, respectively). This proved that the addition of the FAA with a dose of 15 μg was the optimal dose that could add strength to the plasma membrane to protect more sperms from cooling, freezing, and thawing. Based on this condition, the sperms that will be inseminated into the cow’s reproductive tract will be able to survive longer. Given that there was heparin in the female reproductive tract [10], the heparin will induce the FAA receptor on the sperm membrane; thus, signal transduction can take place in the cell, which, in turn, can cause hyperactivation and capacitation [7].

### The effect of FAA on the IPM (%) of bull sperm

The mean (±standard deviation) of the percentage of the IPM of bull sperms post-thawing from various treatments is shown in Table-3. The IPM of a sperm protects the cell for survival. Moreover, it indirectly protects the cell organelles from mechanical and chemical damage.

**Table 3:** Mean (±Standard deviation) and F-test of the percentage of IPM of cow spermatozoa post-thawing on control group and treatment groups added with various doses of FAA.

Treatment	Mean of IPM of spermatozoa (%)±Standard deviation
P^0^	31.57^a^±2.73
P^1^	32.71^a^±2.75
P^2^	36.14^b^±3.28
P^3^	37.25^b^±3.06

P^0^=Control (without being added with FAA), P^1^=Added with FAA 5 μg/200 millions spz progressive motility, P^2^=Added with FAA 10 μg/200 millions spz progressive motility, P^3^=Added with FAA 15 μg/200 millions spz progressive motility. FAA=Fertility-associated antigen, IPM= intact plasma membrane, a and b shows significant difference at p<0.05

The plasma membrane also plays a significant role as a filter for the exchange of intracellular and extracellular substances maintained in the metabolic processes [11]. Damage to the cell membrane will increase its permeability; thus, the materials that should not be allowed to pass through the cell membrane can freely go in and out of the cell and ultimately disrupt the sperm membrane integrity [12].

The results showed that the percentages of the IPM between the groups without and with the additional administration of FAA with a dose of 5 μg did not significantly differ. The additional administration of FAA with a dose of 5 μg was not enough to stabilize the membranes of all sperms; thus, several sperm membranes were damaged because of freezing. When the two groups were compared with those with the additional administration of FAA with doses of 10 and 15 μg, a significant difference was noted. This was because the dose administered was able to stabilize more sperm membranes, resulting in the higher number (percentage) of the IPMs of sperms after freezing.

The percentages of the IPM between the groups with the additional administration of FAA with doses of 10 and 15 μg did not significantly differ (p<0.05). This proved that the addition of the FAA with doses of 10 and 15 μg was the optimal dose that could maintain the stability of the sperm membrane. Thus, at the time of post-thawing, the percentage of the IPM in the group with the additional administration of FAA with a dose of 15 μg was higher than that in the other treatment groups. This was because the dose administered was able to protect more sperm membranes, resulting in the higher number of sperms whose plasma membranes were protected to remain stable; thus, the percentage of sperm membrane damage was lower. Therefore, the dose administered had been able to interact with the plasma membrane, particularly by filling the sockets to increase the density of the sperm membrane. The denser the bond between the components of the membrane, the more stable the membrane will be from the interference originating from extracellular and intracellular substances.

The effect of FAA on the capacitation (%) of bull sperm:

The mean (±standard deviation) of the percentage of capacitation of bull sperm post-thawing from various treatments is shown in Table-4.

**Table 4:** Mean (±Standard deviation) and F-test of the percentage of capacitation of cow spermatozoa post-thawing on control group and treatment groups added with various doses of FAA.

Treatment	Mean of spermatozoa capacitation (%)±Standard deviation
P^0^	23.25^a^±1.20
P^1^	23.12^a,b^±1.45
P^2^	21.87^b,c^±1.14
P^3^	21.05^3^±1.23

P^0^=Control (without being added with FAA), P^1^=Added with FAA 5 μg/200 millions spz progressive motility, P^2^=Added with FAA 10 μg/200 millions spz progressive motility, P^3^=Added with FAA 15 μg/200 millions spz progressive motility. FAA=Fertility-associated antigen, a and b shows significant difference

Capacitation is a process of biochemical reaction and physiological changes in semen before acrosome reaction. During this process, the modification and characterization of the membrane, enzyme activity, and properties of sperm motility [6] as well as a motility change, that is, sperm hyperactivity, occur [13].

Capacitation is a function change observed with an alteration in the Ca^2+^ concentration in the head of a sperm. It can be observed using a technique developed by Mouffok *et al*. [2]. Sperms that have not undergone capacitation Ca^2+^ spreading evenly across their head seem to have yellow fluorescence across the head, whereas those experiencing capacitation Ca^2+^ only on two-thirds of the equatorial region of their head seem to have yellow fluorescence. At the end of capacitation, sperms are characterized by the occurrence of the acrosome reaction, in which the sperm membrane becomes unstable with low Ca^2+^ concentration. Therefore, their head does not have fluorescence, and the fluorescence is only found on the equatorial region of their head. The staining system used was visible fluorescence at the head of the sperms containing Ca^2+^ [14].

During the freezing process, the capacitation percentage significantly decreased (p<0.05) with the addition of the FAA protein in the semen. As shown in Table-4, the groups had the following mean capacitation percentages: Control group without the addition of the FAA protein, 23.25±1.20%; treatment group with the addition of the FAA protein with a dose of 5 μg, 23.12±1.45%; treatment group with the addition of the FAA protein with a dose of 10 μg, 21.87±1.14%; and treatment group with the addition of the FAA protein with a dose of 15 μg, 21.05^c^±1.23%. The percentage of sperm capacitation in the treatment groups with the addition of FAA with doses of 5, 10, and 15 μg decreased. This was because FAA was able to protect the sperm membrane to remain stable so there was no process of capacitation. Sperms undergoing capacitation have an unstable membrane because they are ready to fertilize the oocytes [15].

The group without the addition of FAA showed higher sperm capacitation because after freezing, the plasma membrane of the sperms in this group was damaged and became unstable. Petzoldt and Weitze [16] reported that cooling, freezing, and thawing could cause damage to the plasma membrane. Cell damage caused by cold shock affects the cell structure and function, such as a decrease in the metabolic process of the sperm. The freezing process may cause premature capacitation. This is because the plasma membrane of the sperms is damaged, which causes loss of enzymes required during the capacitation process [7].

The effect of FAA on the pregnancy rate (%) of the cow:

The pregnancy rate after AI with frozen semen added with FAA from various treatments is shown in Table-5. Fertilization is the union of the core of the sperm and ovum nucleus, which is the beginning process of the formation of an individual. The processes directly related to fertilization include capacitation, acrosome reaction of the sperm, fusion of male and female gametes, polysemeny prevention, and completion of meiosis II oocytes [14-16].

**Table 5:** Pregnancy rate of female cow on the control group and treatment groups added with various doses of FAA.

Treatment	Pregnancy rate (%)
P^0^	4 cows (50)
P^1^	4 cows (50)
P^2^	6 cows (75)
P^3^	6 cows (75)

P^0^=Control (without being added with FAA), P^1^=Added with FAA 5 μg/200 millions spz progressive motility, P^2^=Added with FAA 10 μg/200 millions spz progressive motility, P^3^=Added with FAA 15 μg/200 millions spz progressive motility. FAA=Fertility-associated antigen

The factors that play a significant role in the successful implementation of AI are the quality of sperms, inseminator, zootechnics of farmers, and cow. Of all these, the quality of sperms plays the essential role. Fertilization can occur if the sperms have a progressive motility. This enables the sperm to penetrate the egg, but first, it has to undergo capacitation and acrosome reaction [16].

Capacitation speed and acrosome reaction of frozen semen after thawing are strongly influenced by the pH and fluid of the female reproductive tract. The female reproductive tract fluid contains several minerals such as calcium, potassium, magnesium, and sodium chloride as well as glucose and pyruvate as nutritional ingredients for sperms during capacitation [17].

The percentage of successful pregnancy is shown in Table-5. The pregnancy rate in both groups without and with the additional administration of FAA with a dose of 5 μg was 50%, whereas that in the group with the additional administration of FAA with doses of 10 and 15 μg was 75%. Based on the statistical test result, the groups without and with the additional administration of FAA with doses of 5, 10, and 15 μg did not significantly differ. However, the figure showed that the number of sperms added with FAA as much as 10 and 15 μg was higher. It indicated that the addition of FAA could multiply the number of sperms and maintain the stability of the membrane longer in the process of cooling, freezing, and thawing compared with sperms that were not added with FAA. The stability of this membrane occurred because the FAA is a protein that can be attached as a periphery of the plasma membrane especially binding to choline phospholipids; thus, the membrane structure was stronger. Furthermore, FAA binds to glucose to form inhibitor compounds such as glycoprotein containing N-acetylglucosamine, which serves as a protective plasma membrane of sperms to prevent premature capacitation and acrosome reaction. The inhibitor factors block the surface of sperms containing galactosyltransferase enzymes, which can recognize the terminal residues of N-acetylglucosamine on glycoprotein zona pellucida 3 (ZP3) oocytes. A glycoprotein ZP3 oocyte is a ligand (booster) for the sperm for the occurrence of acrosome reaction. In the capacitation process, the inhibitor factor is released; thus, the galactosyltransferase enzyme of sperms is free and able to recognize N-acetylglucosamine on glycoprotein ZP3, leading to binding [14]. Sperms that may undergo capacitation and acrosome reaction are those that have IPM. Integrity of this membrane will be able to perform its function to release enzymes required for the fertilization process.

## Conclusion

The extraction of the accessory sex glands of bulls’ organs from a slaughterhouse should be conducted as soon as possible to avoid denaturation of proteins present in these organs. The addition of FAA in frozen bull semen with a dose of 10 μg/200 jt spermatozoa can improve the fertility of frozen semen by increasing the percentage of viability, motility, and IPM of the sperm post-thawing. Further research on the addition of FAA into bull semen that has substandard quality is required.

## Authors’ Contributions

TWS conceptualized and designed this research. HH conducted the treatments on the samples, freeze bull semen. AI and MH selected the accessory gland organ from slaughterhouse used for samples, data processing, or analysis. FAR conducted extraction, determined molecular weight of FAA using SDS-PAGE method and Western blot. WD conducted electro-elution and measurement of FAA levels using Qubit method. All authors have read and approved the final manuscripts.
